# The Effect of Transmembrane Protein Shape on Surrounding Lipid Domain Formation by Wetting

**DOI:** 10.3390/biom9110729

**Published:** 2019-11-12

**Authors:** Rodion J. Molotkovsky, Timur R. Galimzyanov, Oleg V. Batishchev, Sergey A. Akimov

**Affiliations:** 1 A.N. Frumkin Institute of Physical Chemistry and Electrochemistry, Russian Academy of Sciences, 31/4 Leninskiy Prospekt, 119071 Moscow, Russia; 2 Department of Theoretical Physics and Quantum Technologies, National University of Science and Technology “MISiS”, 4 Leninskiy Prospect, 119049 Moscow, Russia; 3 Moscow Institute of Physics and Technology, 9 Institutskiy Lane, Dolgoprudniy, 141700 Moscow Region, Russia

**Keywords:** liquid-ordered domain, wetting, theory of elasticity, transmembrane protein

## Abstract

Signal transduction through cellular membranes requires the highly specific and coordinated work of specialized proteins. Proper functioning of these proteins is provided by an interplay between them and the lipid environment. Liquid-ordered lipid domains are believed to be important players here, however, it is still unclear whether conditions for a phase separation required for lipid domain formation exist in cellular membranes. Moreover, membrane leaflets are compositionally asymmetric, that could be an obstacle for the formation of symmetric domains spanning the lipid bilayer. We theoretically show that the presence of protein in the membrane leads to the formation of a stable liquid-ordered lipid phase around it by the mechanism of protein wetting by lipids, even in the absence of conditions necessary for the global phase separation in the membrane. Moreover, we show that protein shape plays a crucial role in this process, and protein conformational rearrangement can lead to changes in the size and characteristics of surrounding lipid domains.

## 1. Introduction

The lipid matrix of cellular membranes is quite diverse in composition and contains hundreds of different types of lipids [1]. Fundamental reasons for such diversity are still unclear. For the normal functioning of biological membranes, their lipid bilayer must be in a liquid state. However, the presence of lipids with different temperatures of the main liquid crystalline–gel phase transition can lead to the formation of so-called liquid-ordered domains in a certain temperature range. Lipid molecules in these domains are in the intermediate state between the gel and the liquid [2]. Such domains can be formed in a course of phase separation induced by temperature drop. They have been observed in various model lipid systems and exhibit the following properties: (i) these domains are usually bilayer, i.e., span through the membrane; (ii) their bilayer is thicker than the disordered membrane surrounding them [3]; (iii) these domains are enriched in lipids with saturated hydrocarbon tails with a high liquid crystalline–gel transition temperature, and therefore can be optically observed by using fluorescently labeled saturated lipids [4]. In particular, one of the varieties of such liquid-ordered domains is so-called rafts, lipid domains enriched with sphingolipids and cholesterol [5]. However, while in model systems liquid-ordered domains reach lateral sizes of several micrometers [4,6], such large structures are not observed in cellular membranes. Moreover, even the existence of nanoscopic domains in the membranes of living cells at physiological temperatures is still questioned [7,8,9]. It should be noted that, firstly, the lipid matrix of cellular membranes is asymmetric in the composition of monolayers [10], while micron-sized lipid domains are usually formed in symmetric model lipid systems mimicking the composition of the outer monolayer of the plasma membrane, which is enriched in saturated lipids [11]. Secondly, it is unclear whether conditions for global phase separation in the lipid bilayer of cellular membranes exist at temperatures around 37 °C. In [12], lipid domains of four different compositions were isolated and characterized at the physiological temperature and physiological conditions. In [13] the authors show clustering of sphingomyelin into domains with the size of about 80 nm, although the lateral distribution of cholesterol is uniform. The work [14] demonstrates experimentally observed lipid domains with a size smaller than the diffraction limit of light, although without determining the size itself. However, it is shown in [15,16] using fluorescence microscopy that critical temperature of the lipid extract of the plasma membrane is approximately 25 °C that is significantly lower than the normal physiological temperature. Thus, phase separation in such a membrane at physiological temperature is impossible. In this work, the authors suggest that the bilayer domains of the liquid-ordered phase could either be long-wavelength critical fluctuations of the lipid composition near the critical point [15,16] or occur near condensation (nucleation) centers [17]. The latter option is often justified by the possibility of facilitated compensation of the hydrophobic mismatch between the length of transmembrane regions of various cellular proteins and the thickness of the surrounding membrane due to the increased thickness of liquid-ordered lipid domains [18,19]. A membrane with critical temperature lower than physiological one is called subsaturated under physiological conditions. One of the possible mechanisms of the formation of lipid domains in a subsaturated membrane is the so-called wetting, a local phase transition occurring near the condensation center due to the interaction of membrane components with this center. Membrane proteins may act as condensation centers [18]. This hypothesis is partly consistent with the presence of so-called boundary lipids around proteins that are associated with transmembrane proteins and are extracted with them under the action of detergents [20]. However, the concept of boundary lipids does not explain the formation of large clusters of membrane proteins strictly in the liquid-ordered lipid phase, as well as effect of these domains on many cellular processes, such as signal transmission through cellular membrane [21,22], traffic and sorting of membrane proteins [23,24], cytoskeletal formation [25,26] and viral infection [27].

Generally speaking, under the condition of constant total area of the ordered phase, the characteristic size of lipid domains in equilibrium is determined by the competition of two physical factors: entropy of the ensemble of domains and the energy of the linear boundary between ordered and disordered lipid phases. The entropy factor favors an increase in the number of clusters and, accordingly, a decrease in the size of an individual cluster. The entropic energy contribution depends only on the temperature and it is independent on physical properties of the coexisting phases. The energy of the boundary between domain and its environment contributes to the merging of domains with a decrease in their number and total perimeter, and formation of a macroscopic phase [28,29]. It is assumed that a significant contribution to interfacial energy comes from the difference in thicknesses of the liquid-ordered lipid domain and surrounding membrane, the so-called hydrophobic mismatch [30]. To minimize the contact area of the membrane hydrophobic core with water, the membrane near the boundary should be deformed. This deformation energy determines the magnitude of the boundary energy [31]. To characterize the boundary between domain and surrounding membrane, it is convenient to use the specific energy related to the unit length of the boundary. This specific boundary energy is called line tension. Thus, merging of domains into larger formations or their separation to minimal nanometer-sized lipid clusters is determined by the line tension of the domain boundary [29,32,33].

There are evidences that conformational and functional states of some membrane proteins can be associated with a local change in the phase state of the lipid bilayer around them and the formation of lipid-protein domains (nanoclusters) with the size of several tens of nanometers [34]. Certain lipids mediate clustering of proteins into lipid-protein platforms that differ in properties from the rest of the membrane [35]. In particular, the presence of cardiolipin affects clustering of respiratory proteins in the mitochondrial membrane; violation of this process leads to serious diseases [36].

A well-studied example of transmembrane protein distributed in a liquid-ordered phase of cellular lipid membrane is given by epidermal growth factor receptor (hereinafter—EGFR), belonging to the class of bitopic proteins, receptor tyrosine kinases [37]. The EGFR monomer consists of an extracellular domain that serves as a receptor for the epidermal growth factor (EGF), an intracellular kinase domain triggering a cascade of biochemical reactions in response to the reception of an EGF molecule, a transmembrane domain (TMD) binding these two domains, and juxtamembrane domain located near the inner membrane monolayer [38]. The EGFR dimer [39] is involved in signal transduction; however, the mechanism of dimerization and its relationship with the reception of EGF are still subject of discussion. According to some data, dimerization occurs directly in the process of ligand binding to the extracellular domain [39,40]. An alternative hypothesis suggests that the dimer exists in inactive (closed) and active (open) conformations, and reception of EGF is possible only in the active conformation [38,41]. In this case, the transition of the dimer of the TMD receptor from inactive to active conformation is accompanied by a shift in the connection site of two TMDs from the C-terminus to the N-terminus, i.e., from the inner monolayer of the plasma membrane to the outer monolayer [42]. Moreover, the N-terminal dimerization of EGFR TMD corresponds to an increased ordering of the lipid environment, especially in the inner monolayer of the membrane, where the C-terminus is located [43]. There is evidence of a decisive role of TMD conformational change in signal transmission: the possibility of signal transduction through the membrane without the participation of the membrane in this process is questioned due to the internal disorder of segments of the membrane receptor linker sites [44]. In addition, the functioning of EGFR is modulated by the concentration of important “raft” lipid, cholesterol, in the membrane [45]. It is demonstrated in [46] that the presence of ganglioside GM3 in a phase-separated membrane completely excludes the possibility of EGFR activation. This effect disappears in the absence of phase separation. All these data can be reduced to the assumption of direct involvement of lipid domains in signal transduction by the EGFR [47]. According to this assumption, the dimer of TMDs in the inactive state is predominantly distributed in a liquid-disordered phase. After ligand reception, a liquid-ordered domain is formed near the extracellular domain of EGFR. According to the hypothesis of the lipid-mediated receptor function, formation of the liquid-ordered domain in the inner monolayer leads to the signal transduction through the membrane and triggers a cascade of reactions at the cytoplasmic domain of the receptor [47].

We previously showed that formation of liquid-ordered domains around TMD of proteins can occur by the incomplete wetting mechanism [48]. It manifests in the near-surface formation of the film of a new phase of finite width, with the absence of global phase separation in the membrane [48]. In the current work, we further develop this approach and take into account the compositional asymmetry of the lipid matrix of cellular membranes, as well as the asymmetry of the shape of TMD of proteins. Calculation results allow us to predict the mutual influence of domain formation processes and changes in the conformation of TMD of the EGFR.

## 2. Materials and Methods 

To describe the wetting process, we use the Landau-Lifshitz theory [49]. We consider a subsaturated membrane, i.e., a membrane, in which temperature decrease can lead to the global phase separation resulting in the formation of the ordered domains, but the current temperature is higher than the phase separation temperature of the lipid mixture with the given composition. Quantitatively, subsaturation is defined as a deviation of the current concentrations (or activities) of the membrane components from those at the point of phase transition at the given temperature. In a phase diagram constructed at a fixed temperature, the subsaturation is given by the distance (expressed in terms of the difference in the concentration of lipid components) from the point of the current membrane composition to the phase separation region. To analyze wetting in a multicomponent membrane with *m* being a number of components, we use Slezov model, according to which the composition of the formed ordered phase does not depend on the size of its domain [50]. Formally, this is equivalent to the domain exchanging with the liquid-disordered membrane by not individual lipid molecules, but by some “quasimolecules”, each of which includes all membrane components in the same stoichiometry as the domain. Let a quasimolecule consist of *ν*_i_ (*i* = 1, 2, 3, ... *m*) parts of a lipid molecule of the *i*-th type, so that $\sum_{i=1}^{m}\nu_{i}=1$. The condition of phase equilibrium can be written as follows:

(1) $$\mu_{eq}=\sum_{i=1}^{m}\nu_{i}\mu_{i},$$

where *μ_eq_* is the chemical potential of the quasimolecule in the domain, and *μ_i_* is the chemical potential of the *i*-th component in the liquid-disordered phase. *μ_eq_* can be related to the grand potential of the wetting ordered phase, *W*. It is convenient to use the grand potential in the analysis of the wetting since its independent variables are temperature and chemical potential, which are homogenous throughout the membrane in equilibrium. We denote the total number of quasimolecules in the domain as *n*, the average area per quasimolecule as *a* and the total area of the domain as *s*. Then, according to the well-known thermodynamic relation [49], we obtain:

(2) $$n=-\frac{\partial W}{\partial \mu }=\frac{s}{a}.$$

We normalize the chemical potential to be equal to zero for *s* → ∞. This normalization is chosen solely for convenience since only the difference in chemical potentials has a physical meaning, while an absolute value of the chemical potential at given conditions can be set arbitrary. The infinite increase of the ordered phase area corresponds to the conditions of a global phase separation in the membrane, thus, in a saturated membrane (with zero subsaturation) the chemical potential of quasimolecules is equal to zero. Further, we separately consider cases of the bilayer and monolayer subsaturation. In the first case, the phase separation at a given temperature does not occur in any of the monolayers, i.e., both monolayers of the membrane are subsaturated and their subsaturations are considered equal. The bilayer domain formed in this case is characterized by the total area of the ordered phase *s* in the outer and inner monolayers of the membrane, and radii of the domains in the outer (*R_u_*) and inner (*R_d_*) monolayers are determined by minimizing the free energy of the system. In the case of monolayer wetting, it is assumed that phase separation occurs in one (outer) monolayer of the membrane at a given temperature, i.e., this monolayer is saturated, but in the inner monolayer the global phase separation does not occur, i.e., the inner monolayer is subsaturated. In this case, the state of the system is determined by the radius of the ordered domain *r*, which is formed in the inner monolayer due to the wetting. Thus, in the case of bilayer subsaturation, we consider the dependence *μ*(W(s)), and in the case of monolayer subsaturation, we consider *μ*(*W*(*r*)).

Let us consider the right side of Equation (1). The chemical potentials *μ_i_* can be written as follows:

(3) $$\mu_{i}=k_{B}T\ln\frac{c_{i}}{c_{i}^{eq}},$$

where *c_i_* is the activity of the *i*-th component in the surrounding membrane, $c_{i}^{eq}$ is the equilibrium activity of this component near the boundary of the surrounding membrane and the liquid-ordered domain, *k_B_* is the Boltzmann constant, *T* is the absolute temperature. Difference between activities *c_i_* and $c_{i}^{eq}$ is small near the phase transition, i.e., the subsaturation is small. This allows us to expand Equation (3) in a Taylor series. We keep the first non-zero term in the expansion and substitute the resulting expression into Equation (1). As a result, we get:

(4) $$\mu_{eq}=-k_{B}T\Delta ,$$

where Δ stands for the complete subsaturation; it is determined according to the relation $\Delta =\sum_{i=1}^{m}\nu_{i}\frac{c_{i}^{eq}-c_{i}}{c_{i}^{eq}}$. Equation (4), in particular, indicates that in equilibrium the chemical potential of the domain must be negative.

In accordance with Equation (4), one should define the subsaturation Δ to determine the equilibrium area of the domain. Being a constant, this value is plotted in the graph of *μ*(*s*) or *μ*(*r*) as a straight line parallel to the abscissa for *μ* < 0. The intersection point of this line with the chemical potential plot determines the domain size corresponding to the given subsaturation. The resulting domain might be stable, metastable, or unstable. In the case of several intersection points, we obtain several domain sizes corresponding to the same subsaturation. In order to determine which domain is stable, one should consider the total free energy *E* of the system (i.e., the minimal work necessary to assemble this system). A stable domain corresponds to the global minimum of the free energy *E*, considered as a function of the domain size. This function is defined according to the expression:

(5) $$E=\int \left(\mu +k_{B}T\Delta \right)dn.$$

Utilizing the dependence between *μ* and subsaturation Δ, we calculate the free energy *E* according to Equation (5) and find the equilibrium domain size corresponding to the given subsaturation Δ. The grand potential *W* of the system is determined by the membrane deformation energy arisen from the incorporation of the protein TMD, as well as the energy of deformations due to the compensation of the hydrophobic mismatch at the boundary between the domain and the surrounding membrane. Membrane deformations are considered within the framework of the Hamm–Kozlov model [51]. We introduce the field of unit vectors **n**, called directors, characterizing the average orientation of lipid molecules. This field is related to some surface passing inside the monolayer. The shape of the surface is determined by the field of unit normals **N** to it; normals are considered to be directed towards the intermonolayer surface of the membrane. We take into account two deformation modes—tilt and bending. Deformations and elastic moduli are referred to the so-called neutral surface, on which bending and lateral stretching deformations are energetically independent. Bending deformation is quantitatively described by the divergence of the director along the neutral surface, and tilt deformation is described by the tilt vector **t** = **n**/(**nN**) − **N** ≈ **n** − **N**. We assume that membrane deformations are small. The energy of the deformed monolayer, measured from the state of a flat monolayer, can be represented as [51]:

(6) $$W=\int \left(\frac{B}{2}{\left(\mathrm{div}n\right)}^{2}+\frac{K}{2}t^{2}+\sigma \right)dS-\sigma A_{0},$$

where *B* and *K* are bending and tilt moduli, respectively; σ is the lateral tension of the monolayer; *dS* is the neutral surface area element; *A*_0_ is the neutral surface area in the initial undeformed state. The description of deformations within the framework of such a thermodynamic approach does not allow taking into account any large-scale fluctuations (for example, membrane shape fluctuations), but is focused on the consideration of the membrane deformations in the vicinity of the protein inclusion, at the distances of the order of several nanometers under the assumption of their rapid decay. There are several similar models that describe elastic properties of membranes by introducing deformation modes. They include, for instance, the model proposed by Fournier [52]. In this work, independent elastic moduli of bending, tilt, and tilt variation are introduced and estimated. At the same time, the Hamm-Kozlov model demonstrates that the variation of tilt and bending are additive modes and the contributions of both of them to the elastic energy are determined by the modulus known from measurements of the pure monolayer bending [51]. This allows us to use only two deformation modes, bending and tilt, with bending modulus measured in the experiment, and tilt modulus being estimated from the value of the interfacial tension between water and hydrocarbon tails of lipid molecules. However, it is noteworthy, that in particular cases the elastic models proposed by Fournier and by Hamm and Kozlov yield very similar nontrivial results. For example, the model by Fournier predicts decaying oscillations of the membrane thickness for a certain relation between the elastic moduli [52]. In the framework of the Hamm-Kozlov model the oscillations arises from bending deformation restricted by the condition of local volumetric incompressibility of lipids [31,32,33].

We assume that the protein has a cylindrical symmetry. This allows introducing a cylindrical coordinate system *Ozr* with *Oz* axis coinciding with the axis of symmetry of the protein and *Or* axis lying in a plane perpendicular to *Oz*. The origin of coordinates *O* is located in the plane of the intermonolayer surface of the undeformed membrane. Due to the rotational symmetry, functions determining the shape and deformation of the membrane depend only on the radial coordinate, i.e., the system becomes effectively one-dimensional. In this case, the vector values of the directors, normals to the neutral surface, and tilt vectors can be replaced by their projections onto the *Or* axis: **n** → *n_r_* = *n*, **N** → *N_r_* = *N*, **t** → *t_r_* = *t*. Within the required accuracy, the divergence of the director along the neutral surface can be written in the form: div(**n**) → *dn*(*r*)/*dr* + *n*(*r*)/*r.* In addition, we take into account the condition of local volumetric incompressibility in the following form [51]:

(7) $$h=h_{0}-\frac{h_{0}{}^{2}}{2}{\left(\mathrm{div}n\right)}^{2},$$

where *h* is the thickness of the monolayer at a given point on the neutral surface; *h*_0_ is the thickness of the unperturbed monolayer. The thickness of the unperturbed ordered monolayer is denoted by *h_r_*, and the thickness of the unperturbed monolayer of the surrounding membrane is denoted by *h_s_*. Values related to the outer and inner monolayers are marked by the indices “*a*” and “*b*”, respectively. The shape of the neutral and intermonolayer surfaces *H*(*r*) and *M*(*r*) are described by the distance from the *Or* plane to the point with coordinate *r* on the surface, measured along a perpendicular to the plane *Or*. In such designations the thickness of the outer monolayer is written in the form *h_a_*(*r*) = *H_a_*(*r*) − *M*(*r*), the inner one is given by the equation *h_b_*(r) = *M*(*r*) − *H_b_*(*r*). Within the required accuracy, the radial projection of the normal vector to the neutral surface of the outer and inner monolayers equals to *N_a_* = *dH_a_*/*dr* and *N_b_* = −*dH_b_*/*dr*, respectively. One can express the functions *H_a_*(r) and *H_b_*(*r*) from the Equation (7) in terms of the shape of the intermonolayer surface *M*(*r*) and the radial projections of the directors of the outer and inner monolayers, *a*(*r*) and *b*(*r*). Using the simplified definitions of the tilt vectors *t_a_* = *a* − *N_a_* = *a* − *dH_a_*/*dr*, *t_b_* = *b* − *N_b_* = *b* + *dH_b_*/*dr* and the incompressibility condition (7), we express the tilt vectors in terms of the functions *M*(*r*), *a*(*r*), *b*(*r*) and their derivatives with respect to the coordinate *r*. In addition, we take into account that within the required accuracy:

(8) $$\int \sigma dS-\sigma A_{0}=\int 2\pi r\sigma \left(\sqrt{1+{\left(\frac{dH_{a,b}}{dr}\right)}^{2}}-1\right)dr\approx \int 2\pi r\sigma \frac{1}{2}{\left(\frac{dH_{a,b}}{dr}\right)}^{2},$$

where functions *H_a_*(*r*) and *H_b_*(*r*) can be expressed through *M*(*r*), *a*(*r*), *b*(*r*) and their derivatives with respect to *r*, using Equation (7). We substitute the obtained relations into the elastic energy functional, Equation (6), for each monolayer. The functional of total elastic energy of the membrane equals to the sum of the elastic energies of its monolayers, which depends on three functions: *a*(*r*), *b*(*r*) and *M*(*r*). To find the extremals of this functional, we vary it with respect to the functions *a*(*r*), *b*(*r*), *M*(*r*). As a result, we obtain three Euler-Lagrange differential equations. We substitute the solutions of these equations into the functional of the total elastic energy of the membrane. The expressions for the functions *a*(*r*), *b*(*r*), *M*(*r*) obtained as a result of solving the system of Euler-Lagrange differential equations contain indefinite coefficients, which are determined from the boundary conditions and from the condition of minimum of the total elastic energy. The boundary conditions are determined by the geometry of the TMD of the protein, the requirement of continuity of directors and neutral surfaces, and the requirement of the finiteness of the deformations. The model is described in details in Appendix A.

Our approach based on the Hamm-Kozlov model of lipid membranes elasticity [51] does not include the deformational and compositional coupling terms in the explicit form. However, the volumetric incompressibility condition (7) in this model imposes strong coupling on deformations of membrane leaflets. The function *M*(*r*) is the same for the incompressibility conditions written for the upper and for the lower monolayers, and this imposes ideal balance of forces and torques induced by the monolayers at the intermonolayer surface. From the other hand, the membrane deformations are coupled to the composition of the leaflets via such elastic characteristics as the monolayer equilibrium thickness or elastic moduli. Thus, yet our model does not explicitly include leaflet deformations and composition coupling, it is accounted implicitly via volumetric incompressibility conditions and dependence of the elastic parameters on the lipid composition.

For the quantitative calculations, we use the elastic moduli relevant for the model lipids. Their values in accordance with experimental and theoretical data are taken equal to *B* = 10 *k_B_T*_0_ (*T*_0_ ≈ 300 K is the room temperature, i.e., *k_B_T*_0_ ≈ 4.14⋅10^−21^ J), *K* = 40 mN/m = 10 *k_B_T*_0_/nm^2^ [51,53] for monolayer bending and tilt moduli, respectively. The equilibrium thickness of the ordered monolayer is taken to be equal to *h_r_* = 1.8 nm, the monolayer thickness of the surrounding membrane equals to *h_s_* = 1.3 nm. The lateral tension of the monolayer is taken equal to *σ* = 0.01 *k_B_T*_0_/nm^2^, which is of the same order of magnitude as it is determined for cell plasma membranes [54]. Subsaturation Δ both in the case of a bilayer and in the case of a monolayer wetting is taken to be equal to 1% = 0.01.

## 3. Results

**Figure 1:**
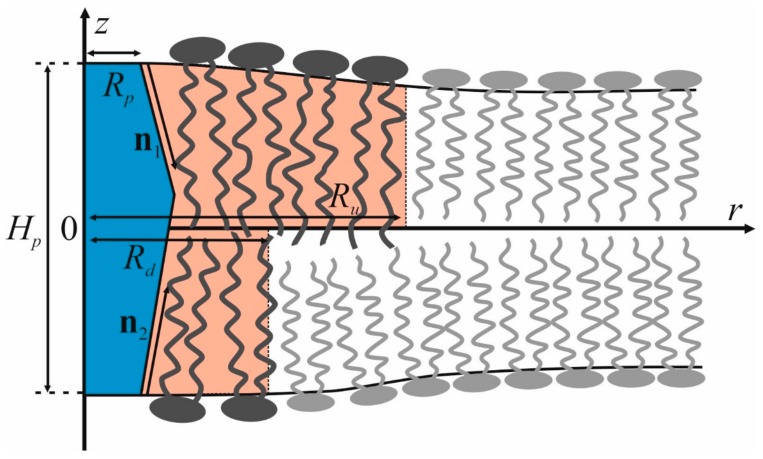
Schematic representation of a lipid-protein domain formed around transmembrane domain (TMD) of the protein by the wetting mechanism. TMD (shown in blue) has the length *H_p_* and the radius in the plane of the membrane *R_p_*. The boundary directors in the upper and lower monolayers are designated as **n**_1_ and **n**_2_, respectively. Liquid-ordered lipid domains in two monolayers of the membrane are highlighted in pink.

We consider the formation of a liquid-ordered lipid domain around a transmembrane protein by the mechanism of wetting. For the sake of generality, we assume that the structure of the lipid domain and the transmembrane domain of the protein does not have mirror symmetry with respect to the membrane midplane (Figure 1). For definiteness, we assume that the membrane is horizontal, with its outer monolayer being in the upper side and the inner one being in the lower side (Figure 1). The state of this system is characterized by five parameters: the radius of the protein, *R_p_*, the radii of ordered domains in the upper and lower monolayers, *R_u_* and *R_d_*, respectively, as well as the radial projections of the boundary directors in the upper and lower monolayers, *n*_1_ and *n*_2_, which can be different. Schematically, this model is presented in Figure 1.

**Figure 2:**
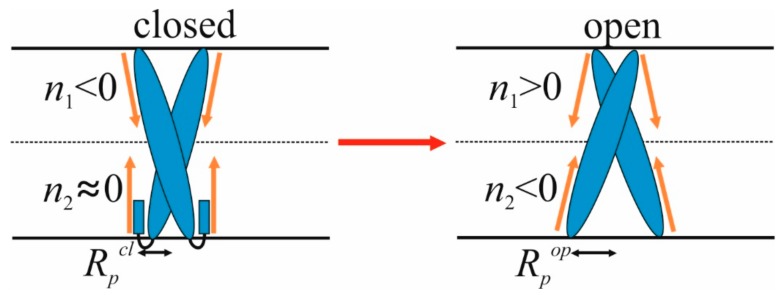
Schematic representation of the conformational transition of TMD of the epidermal growth factor receptor (EGFR) protein from the closed (left) to the open (right) state upon ligand binding. A change in the state of a protein is accompanied by a change in the radius of its TMD, *R_p_*, and radial projections of the boundary directors *n*_1_ and *n*_2_.

We consider the formation of an ordered domain around a transmembrane protein due to wetting in the general case, for arbitrary values of the parameters *R_p_*, *R_u_*, *R_d_*, *n*_1_, *n*_2_. The obtained results are compared with the case of the epidermal growth factor receptor, for which it has been experimentally shown that a change in the conformation and, correspondingly, in the shape of TMDs of its dimer plays a key role in the activation of this receptor [42]. Using these data, we established system parameters that model the EGFR conformational transition from the closed state to the open one. Based on these parameters, we establish the effect of such a change in the shape of the protein on the formation of a liquid-ordered lipid domain in the membrane surrounding the protein. Upon transition from a closed to an open state, the radius *R_p_* increases from approximately 0.9 nm to 1.6 nm [42]. In addition, based on the available data, we can qualitatively assess the change in radial projections of the boundary directors *n*_1_ and *n*_2_. We assume that in the closed state of the receptor *n*_1_ < 0; *n*_2_ ≈ 0, and in the open state *n*_1_ > 0; *n*_2_ < 0 (see Figure 2). The adopted parameter values should be considered as semi-quantitative estimates. Thus, the variation of the radius of TMD by 0.1–0.2 nm should not significantly change the results; the major factor is only a substantial increase in the radius of the TMD when passing from one conformation to another.

We assume that the hydrophobic mismatch is the driving force of wetting, which ensures the predominant interaction of the ordered phase with the protein. For the formation of the ordered domain around the protein under the condition of subsaturation, the length of the TMD should significantly exceed the thickness of the surrounding disordered membrane. In this case, the formation of a thick ordered phase film around the protein can be energetically favorable, and can lead to a local phase transition. Further, for the sake of semi-quantitative analysis, we assume that the length of the TMD, *H_p_*, equals to twice the thickness of the monolayer of the ordered phase, i.e., *H_p_* = 2*h_r_*.

Formation of ordered domains occurs around the room temperature in model membranes mimicking the lipid composition of the outer monolayer of the plasma membrane. This monolayer is enriched in saturated lipids with a high temperature of the main phase transition. The inner monolayer is enriched in unsaturated lipids, and at room temperature usually does not undergo the phase transition [55]. However, according to the data of Förster resonance energy transfer, in a model lipid membrane mimicking the lipid composition of the outer monolayer of plasma membranes, at physiological temperatures, the nanoscopic ordered domains disappear [56]. Thus, it cannot be confidently asserted that at physiological temperature phase separation occurs in the outer monolayer of the plasma membrane. Therefore, we consider two cases: (i) at physiological temperature both monolayers of the membrane are subsaturated (there is no phase separation); (ii) only the inner monolayer is subsaturated, while a domain of the radius *R_u_* is formed in the outer monolayer due to the global phase separation. In the first case, a bilayer ordered domain is formed due to wetting of the protein. In the second case, wetting leads to the formation of the ordered domain in the inner monolayer, while the domain in the outer monolayer preexists as a result of the global phase separation.

**Figure 3:**
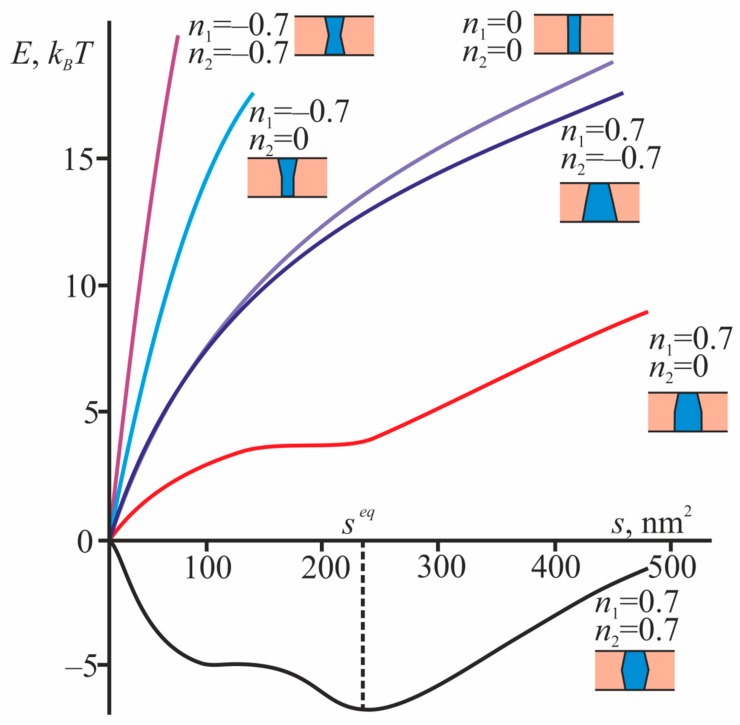
The dependence of the total free energy *E* on the total area of the ordered phase *s* for cases of different shapes of the TMD. The radius of the TMD of the protein is 1.6 nm, which corresponds to the open conformation of the EGFR receptor. For each case, a schematic representation of the corresponding geometry of TMD is given.

First, we consider the possibility of the formation of a bilayer domain. We calculate the dependences of the total free energy *E* on the total area of the ordered phase *s* in two monolayers of the membrane for different radial projections of the boundary directors *n*_1_ and *n*_2_ for the cases of closed and open conformations of EGFR. A typical dependence is shown in Figure 3.

We vary the radial projections of the boundary directors *n*_1_ and *n*_2_ in the range from −0.7 to 0.7. This restriction was introduced for the reason of keeping the deformations small, so that the squared director projection at the TMD boundary was substantially less than unity. For each dependence *E*(*s*), we find the total domain area in the outer and inner monolayers *s_eq_* corresponding to the minimum of the free energy, i.e., equilibrium state of the system. Then we calculate the dependence of the elastic energy *W* on the radius of the domain *R_d_* in the lower monolayer for the fixed total area of the ordered phase around the protein *s_eq_* and find the value of the radius *R_d_* corresponding to the minimum of the energy *W*. The radius of the ordered domain in the upper monolayer *R_u_* is determined from the relation:* s^eq^* = *π*(*R_d_*^2^ − *R_p_*^2^) + *π*(*R_u_*^2^ − *R_p_*^2^). Thus, we determine the size of the domains of the liquid-ordered phase in both monolayers for each given shape of the protein TMD. In this case, a stable ordered domain can be either bilayer (*R_d_*, *R_u_* > *R_p_*) or monolayer (either *R_d_* = *R_p_*, *R_u_* > *R_p_*, or *R_d_* > *R_p_*, *R_u_* = *R_p_*).

**Figure 4:**
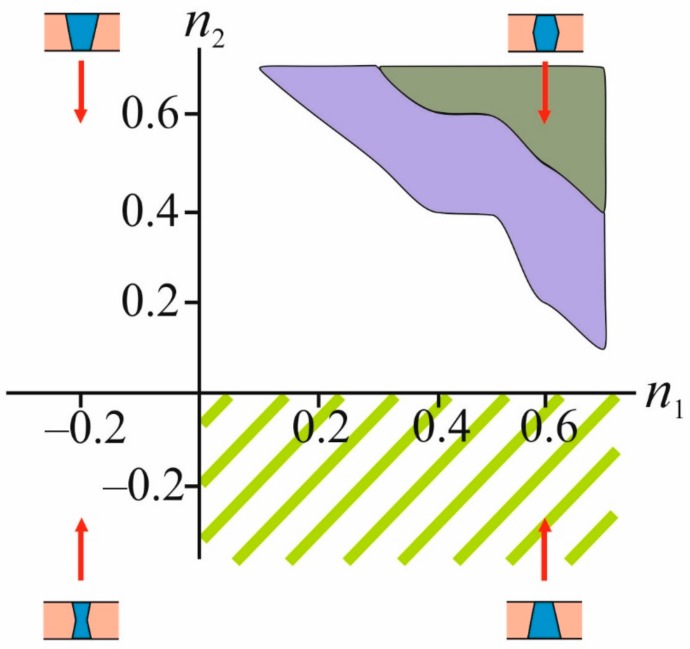
Phase portrait of the system in coordinates (*n*_1_, *n*_2_) in the case of bilayer wetting at *R_p_* = 1.6 nm. The area corresponding to the shape of the TMD of EGFR in the open conformation is highlighted in light green shading. The violet color shades the region of parameters at which the formation of a stable monolayer domain is possible; the green color shades the region of parameters at which the formation of a stable bilayer domain is possible.

We found that in the case of the closed conformation (*R_p_* = 0.9 nm, *n*_1_ < 0; *n*_2_ ≈ 0), stable domains do not form. Moreover, at *R_p_* = 0.9 nm, stable domains are not formed for any physically reasonable values of the radial projections of the boundary directors *n*_1_, *n*_2_. At *R_p_* = 1.6 nm, a stable domain can form around the protein. The domain can be either bilayer or monolayer depending on the specific shape of TMD, i.e., on the value of the radial projections of the boundary directors. However, the range of values (*n*_1_, *n*_2_), at which stable ordered domains are formed, does not intersect with the region (*n*_1_ > 0; *n*_2_ < 0) corresponding to the open EGFR conformation, which is illustrated in Figure 4.

Now we consider the possibility of the formation of a domain in the inner monolayer of the membrane, provided that an ordered domain has been already formed in the outer monolayer as a result of the global phase separation. In addition to the radius of the TMD, we have four geometric parameters that determine the state of the system: the radii *R_u_* and *R_d_* of the domains in the upper and lower monolayers, respectively, and the radial projections of the boundary directors, *n*_1_ and *n*_2_. We varied these parameters for TMD radii of 0.9 and 1.6 nm and determined the sets of parameters at which stable bilayer domains are formed. Calculations carried out for a large number of different sets of parameters *R_u_*, *R_d_*, *n*_1_, *n*_2_ showed that the widths of stable domains in the outer and inner monolayers of the membrane, *L_u_* = *R_u_* − *R_p_* and *L_d_* = *R_d_* − *R_p_*, respectively, very weakly depend on the parameter values and are approximately equal to *L_u_* ≈ 7 nm, *L_d_* ≈ 4 nm. This allows us to fix the domain radii *R_u_* = *R_p_* + 7 nm, *R_d_* = *R_p_* + 4 nm, and analyze their stability at different radial projections of the boundary directors *n*_1_ and *n*_2_. In more detail, the types of dependences of the free energy on the radius of an ordered domain in the inner monolayer, as well as the stability criteria for domains, are presented in Appendix B.

**Figure 5:**
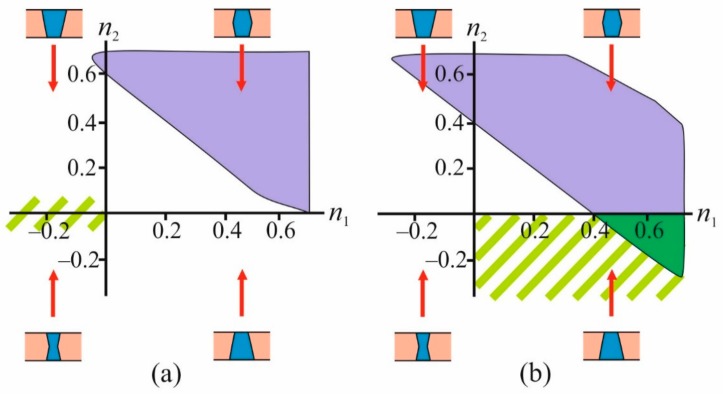
Phase portraits of the system with closed, *R_p_* = 0.9 nm (**a**), and open, *R_p_* = 1.6 nm (**b**), EGFR conformations in (*n*_1_, *n*_2_) plane in the case of monolayer wetting. Light green shading marks the parameter areas (*n*_1_, *n*_2_) that are characteristic of the corresponding conformation. Violet regions indicate the range of parameters at which the formation of the stable domain in the inner monolayer of the membrane is possible. The dark green color indicates the parameter region corresponding to the open conformation of TMD of EGFR, in which the formation of a stable ordered domain in the inner monolayer is possible. The radius of the ordered domain in the outer monolayer was considered equal to *R_u_* = *R_p_* + 7 nm.

By the direct enumeration of various values of the radial projections of the boundary directors (*n*_1_, *n*_2_), we determined the ranges of these parameters in which the formation of a stable ordered domain in the lower monolayer is possible for open and closed conformations of the TMD of EGFR. The radius of the ordered domain in the outer monolayer was considered equal to *R_u_* = *R_p_* + 7 nm. The calculation results are presented in Figure 5.

From the available experimental data [38,42], it can be concluded that for the closed conformation of TMD of EGFR, the characteristic values of the radial projections of the boundary directors should satisfy the relations *n*_1_ < 0; *n*_2_ ≈ 0; for closed conformation, and *n*_1_ > 0; *n*_2_ < 0 for open conformation. Comparing these conditions with the range of parameters (*n*_1_, *n*_2_) under which the formation of a stable ordered domain in the inner monolayer is possible (violet areas in Figure 5), we concluded that the stable ordered bilayer domains do not form in the case of closed conformation of the protein (Figure 5a).

In an open conformation, domains can form in a very narrow range of parameters (*n*_1_, *n*_2_) (the dark green region in Figure 5b). Namely, for the formation of stable domains, the radial projection of the boundary director *n*_1_ in the outer monolayer should be greater than 0.4; in this case, the radial projection of the boundary director in the inner monolayer *n*_2_ should be in the range from 0 to –0.3.

**Figure 6:**
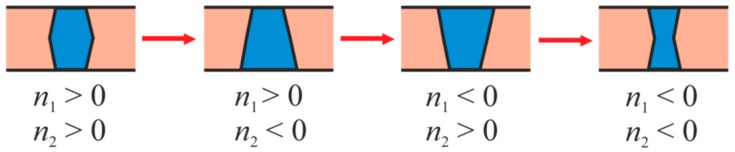
Comparison of the wetting efficiency of TMD of various shapes. Wetting efficiency decreases from the left to the right. The barrel-type TMDs (*n*_1_ > 0, *n*_2_ > 0) are most effectively wetted, the hourglass-type TMDs are least effective (*n*_1_ < 0, *n*_2_ < 0). Conical (*n*_1_ > 0, *n*_2_ < 0) and inverted conical (*n*_1_ < 0, *n*_2_ > 0) TMD are wetted with intermediate efficiency.

Summarizing the results shown in Figure 4 and Figure 5, we present a row of TMD shapes of the same radius corresponding to the decrease of the efficiency of the formation of ordered domains by the wetting mechanism. This row is shown schematically in Figure 6.

## 4. Discussion

In earlier work, we demonstrated the fundamental possibility of forming a liquid-ordered lipid phase film around a transmembrane protein by the mechanism of wetting of protein by the lipid environment [48]. As in the present work, we considered the hydrophobic mismatch between the length of the TMD of the protein and the thickness of the disordered surrounding membrane as a driving force for local phase separation. However, in [48], calculations were performed only for the case of bilayer subsaturation, under the assumption that the resulting domain and membrane as a whole possess mirror symmetry with respect to the intermonolayer surface. The results obtained did not agree with the parameters of real biological systems. Thus, with a subsaturation of Δ = 0.01, a film of a liquid-ordered phase of a width of more than 1 nm was obtained only for TMD radius greater than 5 nm; a film of a width of 5 nm was obtained only for TMD radius exceeding 30 nm. At the same time, for most transmembrane proteins, the TMD radius does not exceed 1–2 nm, and experimental data show that the presence of an ordered phase domain around such proteins is necessary for their normal functioning [12,35,57]. In the present work, we did not require mirror symmetry of the system relative to the intermonolayer surface. It was found that the ordered domains in the outer and inner monolayers have different radii in order to minimize the elastic energy, i.e., the liquid-ordered domain should be asymmetric. This is consistent with the previously theoretically justified asymmetric structure of the domain boundaries of the liquid-ordered lipid phase [58,59]. A similar structure was observed in a number of studies using molecular dynamics methods [60,61,62]. Asymmetry of domains leads to additional relaxation of elastic energy compared to the mirror-symmetric case, due to the “smoothing” of the transition zone between the domain of the liquid-ordered phase and the surrounding membrane. Taking into account the nontrivial structure of the boundary of ordered domains allowed us to obtain adequate sizes of lipid domains formed around TMD of proteins: the equilibrium film width of the ordered phase is ~7 nm for a TMD radius of ~1.5 nm.

Our results also indicate the crucial role of the shape of TMD of the protein, which is the core of the protein-lipid nanocluster. We interpret the change of the conformation of TMD in terms of alteration of its radius and a change of the boundary director projections *n*_1_ and *n*_2_ in two monolayers of the membrane. An increase in the radius of TMD itself should lead to an increase in wetting efficiency due to a decrease in Laplace pressure in the locally formed phase [48]. A comparison of the phase portraits for the open and closed conformations of the TMD of EGFR shown in Figure 5 demonstrate that the radius of the TMD affects, but does not completely determine the wetting process. Indeed, for an open conformation, characterized by a large radius of TMD, with large positive values of the boundary director projections (*n*_1_ = *n*_2_ = 0.6, for instance), a stable bilayer domain does not form (Figure 5b). However, it forms at the same values of *n*_1_ and *n*_2_ in the case of the closed conformation with a smaller radius of TMD (Figure 5a). This observation is reflected in Figure 6, showing the effectiveness of various geometries of protein TMD in terms of domain formation. This means that TMD involved into the signal transduction mediated by the lipid domains, should have similar structure and shape; moreover, small changes in the structure of TMD should lead to critical failures in signal transduction. This prediction of our model finds experimental evidence: it is known that mutation of the N-terminus of certain tyrosine kinase receptors leads to a change in the shape of the TMDs of the receptor dimer, which correlates with the appearance of various pathologies [63,64]. Especially interesting in the light of our results, is the work devoted to the study of the vascular endothelial growth factor receptor 2 (VEGFR-2) [64], which, like EGFR, belongs to the class of bitopic proteins. In this work, the authors build an activation model for the wild-type VEGFR-2. According to their model, upon binding to a ligand, the TMD of the receptor changes its conformation from hourglass to conical geometry. Mutations in TMD of the receptor induced spontaneous activation of the VEGFR-2 in the absence of interaction with the ligand. Thus, TMD of the wild-type VEGFR-2 in the inactive conformation has an hourglass geometry (see Figure 6), and the mutants have a conical or inverted conical shape, approximately corresponding to the shape of the active conformation of the wild type receptor. According to our calculations, this should lead to the preferred formation of wetting lipid domains around mutant TMD that is equivalent to spontaneous activation of the receptor.

In the current work, we did not account for the hydrophobic mismatch between the TMD protein and the liquid-ordered lipid phase (in our calculations it was assumed to be zero), or the spontaneous curvature of the monolayers of the membrane (also taken to be zero), nor the membrane fluctuations. The effect of hydrophobic mismatch on the possibility of the formation of stable domains was previously described in [48] and is trivial from a physical point of view: the greater the hydrophobic mismatch at the boundary with the protein, the more energetically favorable the formation of an ordered domain around this protein. The influence of spontaneous curvature is much less obvious due to its possible different values in the inner and outer monolayers and for the liquid-ordered and liquid-disordered parts of the membrane. This asymmetry can lead to the local deformation of the membrane, as well as to a significant complication of the dependence of the wetting efficiency of the protein on the shape of its TMD and phase diagram of the system [65]. The account for the membrane fluctuations can enhance the wetting, especially in the asymmetric cases [66,67], however, this lies outside our thermodynamical approach. Moreover, the specific lipid components can affect protein-lipid interactions at the protein boundary, as well as protein conformations. These factors will alter the model parameters, first of all, the value of boundary director projections. Therefore, the conclusions made in this work cannot give quantitative predictions on the formation of the ordered domain by wetting for all transmembrane proteins, but they show qualitative trends in the behavior of these protein-lipid structures. In addition, it is necessary to keep in mind the possible effect of the cytoskeleton on TMD, which limits the size of the lipid system around TMD and increases the likelihood of a lipid-protein nanocluster formation [68]. This can also be taken into account in the framework of our model by introducing appropriate boundary conditions and varying the surface tension in different parts of the membrane.

## 5. Conclusions

In summary, in the present work, we developed a model describing the formation of liquid-ordered domain around transmembrane proteins in subsaturated membrane, i.e., in the absence of conditions for the global phase separation. As the driving force of the local phase separation, we considered a hydrophobic mismatch between the length of the protein transmembrane domain and the thickness of the liquid-disordered bilayer. For relatively long TMD it was energetically favorable to surround the protein by the liquid-ordered domain. The thickness of the ordered bilayer is larger than that of the surrounding disordered membrane. Thus, in the process of wetting in such system, the hydrophobic mismatch between undeformable (infinitely rigid) protein and the disordered membrane is effectively transformed to the thickness mismatch between deformable (i.e., softer) ordered and disordered lipid phases.

It was shown that the formation of stable liquid-ordered domain depends on the TMD radius. Generally, larger radius of TMD promotes wetting. Besides, the wetting effectiveness is strongly dependent on the TMD shape, which is parameterized by radial projections of boundary directors. Generally, the wetting is most effective when the projections of the boundary directors of the outer and inner monolayers are both positive (Figure 4 and Figure 5), i.e., when the TMD has a barrel-like shape. Thus, in order to induce the signal, which demands formation of the membrane-spanning ordered domain, the protein TMD should be able to change its conformation from inactive (small radius, negative or only slightly positive radial projections of boundary directors) to the active one (larger radius, positive radial projections of boundary directors) upon binding with the ligand. This conclusion can be drawn for the membrane, in which one or both monolayers are subsaturated.

## Figures and Tables

**Figure A1:**
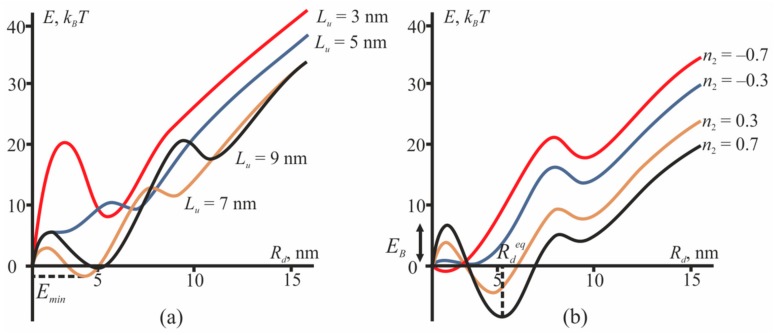
The dependence of the total free energy *E* on the radius *r* of the domain in the inner monolayer. (**a**) For a fixed domain width in the outer monolayer *L_u_* = 3, 5, 7, 9 nm. The remaining parameters were considered equal to *R_p_* = 1 nm, *n*_1_ = 0.5, *n*_2_ = 0.5. (**b**) For a fixed radial projection of the boundary director in the inner monolayer *n*_2_ = −0.7, −0.3, 0.3, 0.7. The remaining parameters were considered equal to *R_p_* = 1.6 nm, *L_u_* = 7 nm, *n*_1_ = 0.6.

## Appendix A

Bilayer Domain Formation

Consider a membrane consisting of *m* lipid components, in which there is a cylindrically symmetric TMD protein of radius *R_p_*. We assume that both monolayers of the membrane are subsaturated; in this case, ordered domains formed in different monolayers can have different sizes. We assume that the lipid composition of a domain does not depend on its size. This condition equivalent to that the size of the domain changes due to the exchange with the liquid-disordered surrounding membrane by “quasimolecules”, each of which including the lipid components of the membrane in the same stoichiometry as the whole domain. Let the quasimolecule include the fraction *ν_i_* (*i* = 1, 2, 3, ... *m*) of the lipid molecule of each of the *m* lipid components, so that that $\sum_{i=1}^{m}\nu_{i}=1$. Then the change in the free energy of the system upon the addition of one quasimolecule to a domain reads as follows:

(A1) $$\Delta \mu =\mu \left(s\right)-\sum_{i=1}^{m}\nu_{i}\mu_{i},$$

where *s* is the domain area; *μ*(*s*) is the chemical potential of the quasimolecule in the domain, and *μ_i_* is the chemical potential of the *i*-th component in the liquid-disordered surrounding membrane. Under the equilibrium conditions Δ*μ* = 0, i.e.:

(A2) $$\mu \left(s_{e}\right)=\sum_{i=1}^{m}\nu_{i}\mu_{i},$$

where *s_e_* is the equilibrium domain area. The chemical potential *μ*(*s*) of the quasimolecules in the domain can be associated with the large canonical potential of the membrane *W*. If *n* is the number of quasimolecules in the domain, then:

(A3) $$n=-\frac{\partial W}{\partial \mu }.$$

We denote the average area per quasimolecule by *a*. Then Equation (A3) can be rewritten as follows:

(A4) $$\frac{s}{a}=n=-\frac{\partial W\left(s\right)}{\partial \mu \left(s\right)}=-\left(\frac{\partial W\left(s\right)}{\partial s}\right)/\left(\frac{\partial \mu \left(s\right)}{\partial s}\right).$$

Phase separation conditions demand formation of the domain of infinite radius at zero subsaturation. That means that at *s* → ∞ the chemical potential should equal to zero. Expression (A4) can be converted to the following form:

(A5) $$\mu \left(s\right)=\int_{s}^{\infty }\frac{a}{x}\frac{\partial W\left(x\right)}{\partial x}dx.$$

Consider the right side of Equation (A2). The chemical potentials *μ_i_* can be written as follows:

(A6) $$\mu_{i}=k_{B}T\ln\frac{c_{i}}{c_{i}^{eq}},$$

where *c_i_* is the activity of the *i*-th component in the surrounding membrane, $c_{i}^{eq}$ is the equilibrium activity of this component in the presence of a liquid-ordered domain in the membrane. Near the phase transition, the activity *c_i_* and $c_{i}^{eq}$ do not differ much from each other, i.e., the subsaturation is small. This allows expanding the expression (A6) into a Taylor series by subsaturation; keeping the first nonzero term in the expansion, we get:

(A7) $$\mu_{i}=k_{B}T\frac{c_{i}-c_{i}^{eq}}{c_{i}^{eq}}.$$

Substitution of expression (A7) into (A2) gives:

(A8) $$\mu \left(s_{e}\right)=-k_{B}T\sum_{i=1}^{m}\nu_{i}\frac{c_{i}^{eq}-c_{i}}{c_{i}^{eq}}.$$

The expression under the sum sign is the total subsaturation. We denote it by Δ:

(A9) $$\Delta =\sum_{i=1}^{m}\nu_{i}\frac{c_{i}^{eq}-c_{i}}{c_{i}^{eq}}.$$

It follows from definition (A9) that Δ is a positive quantity. Taking into account equalities (A5) and (A9), the expression (A8) can be rewritten in the following form:

(A10) $$\int_{s}^{\infty }\frac{a}{x}\frac{\partial W\left(x\right)}{\partial x}dx=-k_{B}T\Delta .$$

It is seen from the Equation (A10) that in equilibrium the chemical potential of the domain must be negative.

In accordance with expression (A10), to determine the equilibrium area of an ordered domain, one needs to define the value of the subsaturation Δ. This value is independent of *s* and therefore can be plotted as a straight line parallel to the abscissa axis on the graph of *μ*(*s*). The intersection point of this line with the graph *μ*(*s*) determines the area of the domain corresponding to this subsaturation. Moreover, such a domain can be stable, metastable or unstable. In the case when there are several intersection points, we get several domains corresponding to the same subsaturation. In order to determine which one is stable, we must analyze the total free energy *E* of the system. A stable domain corresponds to the global minimum of free energy *E*(*s*) as a function of the area of the domain. This function is defined according to the expression:

(A11) $$E\left(s\right)=\int_{s_{p}}^{s}\left(\mu \left(s\right)+k_{B}T\Delta \right)dn,$$

where *s_p_* is the cross-sectional area of TMD of the protein. Substituting the expression *dn *= *ds/a* into Equation (A11), we obtain:

(A12) $$E\left(s\right)=\frac{1}{a}\int_{s_{p}}^{s}\left(\mu \left(s\right)+k_{B}T\Delta \right)ds.$$

For a given *μ*(*s*) and subsaturation Δ, we can calculate the free energy of the system *E*(*s*) according to the Equation (A12) and find the equilibrium domain area corresponding to this subsaturation Δ.

Monolayer Domain Formation

Let us consider the case when the phase separation in the outer monolayer has already occurred, i.e., its subsaturation equals to zero, while the internal monolayer is subsaturated. All values should depend only on the radius of the domain *r* in the inner monolayer. The dependence of the chemical potential on the radius of the domain in the inner monolayer has the following form:

(A13) $$\mu \left(r\right)=\int_{r}^{\infty }\frac{a}{\pi \left(x^{2}-R_{p}{}^{2}\right)}\frac{\partial W\left(x\right)}{\partial x}dx,$$

where *R_p_* is the radius of the transmembrane domain of the protein. The expression (A10), which relates the chemical potential to the subsaturation, changes accordingly:

(A14) $$\int_{r}^{\infty }\frac{a}{\pi \left(x^{2}-R_{p}{}^{2}\right)}\frac{\partial W\left(x\right)}{\partial x}dx=-k_{B}T\Delta .$$

The total free energy *E* depends on the radius of the domain *R_d_* in the inner monolayer. It can be represented as follows:

(A15) $$E\left(R_{d}\right)=\int_{R_{p}}^{R_{d}}\left(\mu \left(r\right)+k_{B}T\Delta \right)dn.$$

Substituting the expression $dn=\frac{2\pi rdr}{a}$ into (A15), we obtain the free energy of the system:

(A16) $$E\left(R_{d}\right)=\frac{1}{a}\int_{R_{p}}^{R_{d}}2\pi r\left(\mu \left(r\right)+k_{B}T\Delta \right)dr.$$

Boundary Conditions

The state of the membrane is characterized by five functions: the shape of the neutral surface of the outer monolayer *H_a_*(*r*), the inner monolayer *H_b_*(*r*), the intermonolayer surface *M*(*r*), the radial projection of the director in the outer monolayer *a*(*r*) and the radial projection of the director in the inner monolayer *b*(*r*). Using the conditions of local volume incompressibility, the shapes of the neutral surfaces of monolayers are expressed through the director projections *a*(*r*), *b*(*r*) and the shape of the intermonolayer surface *M*(*r*). Variation of the functional of elastic energy, Equation (6), with respect to these functions leads to three Euler-Lagrange differential equations. Their solutions *a*(*r*), *b*(*r*), *M*(*r*) contain indefinite coefficients, which are determined from the boundary conditions. These conditions are implied at the protein boundary (*r* = *R_p_*), at the boundary of the ordered domain with the surrounding membrane (*r* = *R_u_*, *r* = *R_d_*). The boundary conditions at the protein are determined by the TMD shape. The latter is determined by its length *H_p_*, as well as the radial projections of the boundary directors *n*_1_ and *n*_2_ in the upper and lower monolayers, respectively (see Figure 1); in this case, *n*_1_ may be unequal to *n*_2_, which models the possible asymmetry of TMD of the protein. The boundary conditions have the following form:

(A17) $$a\left(R_{p}\right)=n_{1};\;\;\;b\left(R_{p}\right)=n_{2}.$$

In addition, we assume that the length of TMD, *H_p_*, coincides with the thickness of the unperturbed ordered bilayer 2*h_r_*:

(A18) $$H_{a}\left(R_{p}\right)-H_{b}\left(R_{p}\right)=H_{p}=2h_{r}.$$

At the boundary between the ordered domain and the surrounding membrane, we impose conditions of the continuity of radial projections of directors and neutral surfaces. In addition, far from the protein (*r* → ∞), the membrane is assumed to be unperturbed, i.e., the radial projections of the directors are considered equal to zero, the neutral surfaces and the intermonolayer surface are considered horizontal, the thicknesses of each monolayer are considered equal to the thickness of the unperturbed monolayer of the liquid-disordered phase. Thus, we have the boundary conditions in the following form:

(A19) $$a\left(\infty \right)=0;\;\;\;b\left(\infty \right)=0;\;\;\;H_{a}\left(\infty \right)=h_{s};\;\;\;H_{b}\left(\infty \right)=-h_{s};\;\;\;M\left(\infty \right)=0.$$

These boundary conditions make it possible to determine some of the uncertain coefficients emerging in the solutions of the Euler-Lagrange equations. To find the remaining coefficients, the solutions are substituted into the elastic energy functional, and the elastic energy is minimized with respect to the uncertain coefficients. All expressions were obtained analytically, but we do not provide them because of their extreme bulkiness. The methodology for finding elastic energy is described in more detail in [69,70,71].

## Appendix B

Let us consider the case of the membrane with the subsaturated inner monolayer and ordered domains of radius *R_u_* in the outer monolayer. The membrane state depends on four variable parameters—the radii of the domains *R_u_*, *R_d_* and the radial projections of the boundary directors *n*_1_, *n*_2_ in the outer and inner monolayers, respectively. Our goal is to determine the range of parameters at which a stable ordered domain can form in the inner subsaturated monolayer. For this, we calculate the dependence of the total free energy *E* on the radius of the domain *r* in the inner monolayer for various sets of three parameters: *L_u_* (*L_u_* = *R_u_* − *R_p_*), *n*_1_ and *n*_2_. A typical resulting dependency is shown in Figure A1.

A minimum in the dependence *E*(*r*) corresponds to a stable domain, moreover, the free energy *E_min_* in the minimum should be less than the energy in the initial state, at *r* = 0. As can be seen from Figure A1b, there are two types of dependence *E*(*r*) for which the formation of a stable domain is possible. In the first case, the free energy monotonically decreases down to the minimum point and then increases (the red curve corresponding to *n*_2_ = −0.7). In the second case, there is an energy barrier between the global minimum point and the initial state *r* = 0. Our calculations show that the barrier-free dependences *E*(*r*) correspond to the width of the equilibrium domain in the inner monolayer of approximately 1 nm, i.e., one layer of lipids. Such cases are excluded from consideration since the boundary layer of lipids cannot be considered as a phase and cannot be considered within the framework of continuum models. Then we consider only the dependences *E*(*r*) exhibiting the energy barrier. We select those dependencies for which two conditions are satisfied: (i) the resulting domain is stable, i.e., *E_min_* < 0; (ii) the energy barrier *E_B_* of the formation of the equilibrium domain is relatively small (less than 5 *k_B_T*).

Our calculations show that for the fixed values of the parameters *n*_1_ and *n*_2_, stable domains in the inner monolayer correspond to a very narrow range of domain widths *L_u_* in the outer monolayer. Therefore, according to Figure A1a, the minimum free energy with a relatively small initial energy barrier is achieved at *L_u_* = 7 nm. This value weakly depends on the values of the remaining parameters. Therefore, in further calculations, we fixed *L_u_* = 7 nm.
